# Updates on the molecular spectrum of *MEFV* variants in lebanese patients with Familial Mediterranean Fever

**DOI:** 10.3389/fgene.2024.1506656

**Published:** 2025-01-17

**Authors:** Rudy Feghali, José-Noel Ibrahim, Nabiha Salem, Romy Moussallem, Ghina Hijazi, Charbel Attieh, Tony Yammine, Alain Chebly

**Affiliations:** ^1^ Faculty of Medicine, Saint Joseph University of Beirut, Beirut, Lebanon; ^2^ Department of Biological Sciences, School of Arts and Sciences, Lebanese American University (LAU), Beirut, Lebanon; ^3^ Center Jacques Loiselet for Medical Genetics and Genomics (CGGM), Faculty of Medicine, Saint Joseph University of Beirut (USJ), Beirut, Lebanon

**Keywords:** Familial Mediterranean Fever, FMF, genotype-phenotype correlation, recurrent fever, MEFV, variants, Lebanon

## Abstract

Familial Mediterranean Fever (FMF) is a hereditary autoinflammatory disorder, particularly present in the Mediterranean populations, and associated with pathogenic variants in the *MEFV* gene. This study aims to investigate the distribution of *MEFV* variants in a large cohort of Lebanese patients, and to explore the genotype-phenotype correlation among affected individuals. A retrospective analysis was conducted on 3,167 patients referred for *MEFV* sequencing at the Medical Genetics and Genomics Center(CGGM) at Saint-Joseph University of Beirut-Lebanon, from 2006 to 2023. Sanger sequencing was used to detect *MEFV* variants, focusing initially on hot-spot exons. Among the 3,167 patients, 46.73% (N = 1,480) carried at least one *MEFV* variant. The most common variants detected were M694V and V726A, both found in 28.98% of cases, followed by E148Q(27.83%) and M694I(13.98%). Moreover, Shiites and Sunni Muslims, and individuals from South and North Lebanon exhibited the highest frequency of variants. Interestingly, family history was found to be significantly higher in patients having two *MEFV* variants than those with one variant (*p* = 0.0026). The most commonly reported symptoms were fever(78%), abdominal pain(88%), joint pain(65%), and thoracic pain(46%). The genotype-phenotype correlation analysis revealed a more severe phenotype in patients carrying the M694V or V726A mutations compared to those with the homozygous E148Q genotype. This study, the largest in Lebanon, highlights the high prevalence of *MEFV* variants, particularly M694V and V726A, in FMF patients. Our data provide valuable insights into the genetic landscape of FMF in Lebanon and emphasize the importance of early genetic screening for a better disease management and genetic counselling.

## Introduction

Familial Mediterranean Fever (FMF) (MIM # 249100) is a hereditary autoinflammatory disorder predominantly affecting populations around the Mediterranean Sea including Arab and non-Arab countries. The disease is reportedly less common in other ethnic groups and populations, but it is becoming more prevalent worldwide due to global population movements (Lancieri et al., 2023).

FMF is mainly inherited in an autosomal recessive manner and caused by pathogenic variants in the Mediterranean Fever (*MEFV*) gene located on chromosome 16p13.3. This gene comprises 10 exons, encoding a 781-amino acid protein, named pyrin, that regulates inflammation. Mutations in *MEFV* lead to the loss of pyrin’s regulatory function, resulting in uncontrolled inflammation. Indeed, abnormal pyrin interacts with the inflammasome, a multiprotein complex responsible for activating pro-inflammatory cytokines, mainly interleukin-1β (IL-1β) and IL-18 (TUFAN and LACHMANN, 2020; Ibrahim et al., 2017; Ibrahim et al., 2018; Ibrahim et al., 2014). To date, around 400 *MEFV* variants have been identified (ClinVar, 2024; Infevers, 2024), with exon 10 and exon 2 being considered as hotspots for pathogenic variants (Bernot et al., 1998), and M694V being the most frequent mutation detected in FMF patients worldwide (Touitou, 2001).

FMF symptoms typically start before adulthood, with 90% of patients experiencing their first attack before the age of 20. The hallmark of FMF is recurrent, self-limiting febrile episodes accompanied by serositis. These attacks usually last between 1 and 3 days and manifest by symptoms like fever with chills (resolving within 24–72 h), abdominal pain, chest pain (pleural serositis), and joint pain (arthralgia or arthritis). Amyloidosis is the most severe complication of FMF, resulting from the deposition of serum amyloid A (SAA) protein in various organs, particularly in the kidneys. If not properly managed, it can lead to severe consequences including renal failure (Siligato et al., 2021). Interestingly, the wide variability in disease expression among individuals is primarily related to the allelic heterogeneity of *MEFV*, but is also influenced by other factors, including modifying genes and epigenetic events (Cazeneuve et al., 2000; Ibrahim et al., 2015; Chaaban et al., 2024a).

The diagnosis of FMF is primarily based on typical clinical findings in association with probable ethnicity, family history, and in some cases response to colchicine. Genetic testing is used to confirm the clinical diagnosis of FMF. It is very important for good clinical management, and to offer genetic counselling for FMF patients and their families, particularly in atypical cases or in populations with low disease prevalence (Manna and Rigante, 2019).

The gold standard treatment of FMF is the daily administration of colchicine, which significantly reduces the frequency and severity of attacks and prevents amyloidosis. This medication works by inhibiting microtubule polymerization, thereby reducing leukocyte chemotaxis and the inflammatory response. It is also thought to inhibit NF-κb signaling pathway and pyrin inflammasome assembly (Mezher et al., 2024). It is reported to be effective in 60%–75% of FMF patients, with partial response in an additional 10%–20%. The standard dosage varies from 0.5 to 2.0 mg/day, and it can be adjusted based on patient response and tolerance (Ozen et al., 2016; Ozturk et al., 2011). For patients unresponsive to colchicine or those experiencing severe side effects, alternative treatments include biologic agents targeting interleukin-1 (IL-1) such anakinra and canakinumab that are approved by the EMA (European Medicines Agency) and the FDA (Food and Drug Administration), respectively (Eroglu et al., 2015; Chaaban et al., 2024b).

In the Arab world, conducting FMF-related research is minimal, counting for only 3.80% of the total FMF-related publications worldwide (Masri et al., 2022). Even though Lebanon ranks first in FMF articles’ contribution among Arab countries (after normalizing to average population size, GDP, and number of physicians), there is still a lot of aspects to be addressed (Assouad et al., 2021). In Lebanon, the investigation of FMF started in 2005 with the analysis of specific *MEFV* gene variants (M694V, M680I, V726A, M694I, and E148Q) in a small cohort of patients (Medlej-Hashim et al., 2005; Sabbagh et al., 2008). This initial study identified M694V as the most prevalent variant, aligning with global patterns (Medlej-Hashim et al., 2005). Subsequent haplotype analysis across different Lebanese religious groups (Shiite, Sunni Muslims, and Christians) revealed that these variants originated from an ancient common ancestral population, where most *MEFV* mutations were already present with their associated haplotypes (Jalkh et al., 2008). Recently, a study done in Southern Lebanon examined 23 *MEFV* variants in 332 clinically diagnosed FMF patients. The findings supported the previous distribution of variant frequencies and highlighted the role of pseudo-dominant transmission of the disease in this region (El Roz et al., 2020). Despite providing valuable insights, these studies present limitations that are mainly related to the relatively sample size and the restricted number of tested *MEFV* variants, hence reducing the generalizability of the results. These limitations underscore the necessity to have a more comprehensive analysis of *MEFV* variants spectrum by looking at rare and newly identified variants and by analyzing those of uncertain significance (VUS) on a larger sample.

Accordingly, the current study is designed to analyze the molecular aspects of FMF and the spectrum of *MEFV* variants in the largest series of patients belonging to different geographic, ethnic, and religious groups across Lebanon. It also aims to explore the genotype-phenotype correlation of the most three common *MEFV* variants among affected individuals. The purpose is to establish a more accurate diagnosis of FMF, ultimately enabling improved management and treatment of the disease.

## Materials and methods

### Study design and population

This is a retrospective study on *MEFV* gene testing between 2006 and December 2023. A total of 3,167 clinically diagnosed FMF patients were referred, by either general physicians or specialists, to the Jacques Loiselet Center for Medical Genetics and Genomics (CGGM) at Saint Joseph University of Beirut (USJ) in Lebanon for *MEFV* sequencing. The major reported clinical symptoms were fever, abdominal pain, joint pain, thoracic pain and diarrhea. This study was conducted according to the Declaration of Helsinki and approved by the Ethical Committee at Saint Joseph University of Beirut and Hotel Dieu de France in Beirut, Lebanon (CEHDF-2315). An informed consent was obtained from all the participants prior to enrollment.

### MEFV sequencing

A blood sample was collected from all participants in EDTA tubes. DNA was extracted either by salting out method or using the QIAamp DNA^®^ Kit (Qiagen). DNA quantity and quality were assessed by spectrophotometry using the Nanodrop ND-1000.

PCR reactions were performed using specific sets of primers (Forward and Reverse) for each exon. Exon 10 was sequenced first, followed by exons 2, 3, and 5 if needed. The remaining exons are tested upon recommendation to search for the second *MEFV* variant in heterozygous individuals. Then, PCR products were run on 1% agarose gel for verification. Afterwards, PCR products were purified and then sequenced using the Big Dye Terminator v1.1 Cycle sequencing kit (Applied Biosystems) under standard conditions. Sequencing reactions were purified using Sephadex G50 (Amersham Pharmacia Biotech) and loaded into the ABI 3130 or 3,500 Sequencer for capillary electrophoresis. Obtained electropherograms were analyzed and compared to the reference sequences using ChromasPro software (Technelysium).

## Results

**FIGURE 1:**
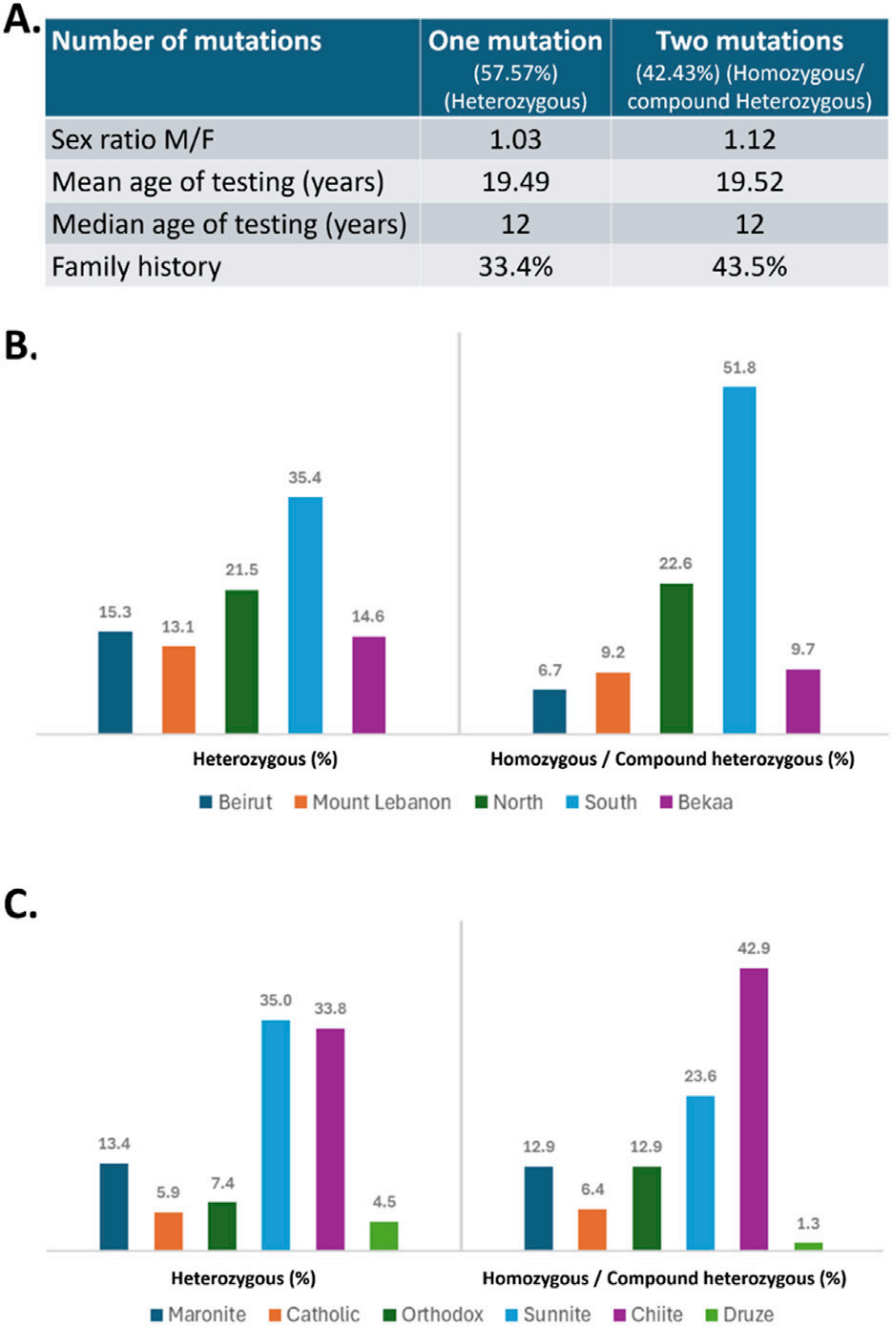
**(A)** Demographic characteristics of the studied population. **(B)** Geographic distribution (in %) of patients with one mutation (heterozygous) or two mutations (homozygous/compound heterozygous) across different regions of Lebanon. **(C)** Religious distribution (in %) in both mutation groups. Chi square analysis revealed a significant greater percentage of family history among patients carrying two mutations compared to those with one mutation (*p* = 0.0026).

Among the 3,167 patients tested for *MEFV* variants, a total of 1,480 (46.73%) patients showed positive results, presenting at least one *MEFV* variant. Out of the 1,480 patients presenting variations in the *MEFV* gene, 48.38% were females and 51.62% were males, with an M/F sex ratio equal to 1.03 in the “heterozygous” group and 1.12 in patients carrying two variants (homozygous or compound heterozygous). In 64.88% of cases (n = 728), the age at testing was before 20 years old, with a mean age and a median age of 19 years and 12 years, respectively, in both groups (Figure 1). Of the 1,480 patients presenting *MEFV* variants, 628 patients (42.43%) presented two mutated alleles, with 177 (28,18%) being homozygous and 451 (71,82%) compound heterozygous (Table 1). The remaining 852 patients (57.57%) showed only one mutated allele and were classified as heterozygous. Interestingly, our findings revealed a significantly greater percentage of family history of FMF among patients carrying two *MEFV* variants (43.5%) compared to those with one variant (33.4%) (*p* = 0.0026).

**TABLE 1:** Frequency of the identified *MEFV* variants among heterozygous, homozygous, and compound heterozygous patients.

Status (N = 1,480)	Genotype	Number	% Among N1, N2, or N3	% of total (N = 1,480)
Heterozygous (N1 = 852) 57.57%	E148Q M694V V726A M694I A744S R761H M680I P369S K695R A289V Other	267 166 144 72 64 25 21 15 14 10 54	31.34% 19.48% 16.90% 8.45% 7.51% 2.93% 2.46% 1.76% 1.64% 1.17% 6.36%	18.04% 11.22% 9.73% 4.86% 4.32% 1.69% 1.42% 1.01% 0.96% 0.68% 3.65%
Homozygous (N2 = 177) 11.96%	M694V V726A M694I M680I E148Q R761H A744S P369S	65 48 29 18 9 6 1 1	36.72% 27.12% 16.38% 10.17% 5.00% 3.39% 0.56% 0.56%	4.39% 3.24% 1.96% 1.22% 0.60% 0.41% 0.07% 0.07%
Compound Heterozygous (N3 = 451) 30.47%	M694V/V726A M694I/V726A E148Q/M694V E148Q/V726A M680I/V726A M694I/M694V M694I/E148Q M694V/R761H M680I/M694V P369S/R408Q E148Q/M680I M694I/R761H V726A/R761H Other combinations Complex Genotypes	92 46 40 31 26 21 20 15 12 11 10 10 9 108 20	20.40% 10.20% 8.87% 6.87% 5.76% 4.66% 4.43% 3.33% 2.66% 2.44% 2.22% 2.22% 2.00% 23.95% 4.64%	6.22% 3.10% 2.70% 2.09% 1.76% 1.42% 1.35% 1.01% 0.81% 0.74% 0.68% 0.68% 0.61% 7.30% 1.35%

On the other hand, most of the patients, heterozygous or homozygous/compound heterozygous, originated from South Lebanon and North Lebanon, with fewer cases from other regions including Beirut, Mount Lebanon, and Bekaa (Figure 1). A more detailed analysis of religious distribution revealed that the majority of the patients were Muslim Shiite or Sunni, while Druze patients were the minority (Figure 1).

In this study, we identified 48 different variations in the *MEFV* gene among 1,480 Lebanese patients. The distribution of the most prevalent variants and their allele frequencies are represented in Table 2. M694V and V726A were the most commonly detected variants in the studied population, accounting each for 28.98% of the cases, followed by E148Q (27.83%) and M694I (13.98%). M680I, A744S, R761H, and P369S accounted for 6.42%, 5.54%, 4.93%, and 3.04% respectively. On the other hand, M694V presented the highest allele frequency (16.57%), followed by V726A (15.99%), E148Q (14.22%), M694I (7.91%), M680I (3.79%), A744S (2.78%), R761H (2.65%), and finally P369S (1.54%). Interestingly, a significant number of patients (N = 178 or 12%) showed the presence of other variants that are considered rare or not very frequent.

**TABLE 2:** Distribution of *MEFV* variants and allele frequencies among participants (N = 1,480).

Variant	Exon	Variant classification		Variant frequency N (%)	Allele frequency N (%)
		Infevers	ClinVar		
M694V	Exon 10	Pathogenic	Pathogenic/Likely Pathogenic	429 (28.99%)	494 (16.57%)
V726A	Exon 10	Pathogenic	Pathogenic/Likely Pathogenic	429 (28.99%)	477 (15.99%)
E148Q	Exon 2	VUS	Conflicting classifications of pathogenicity	412 (27.83%)	424 (14.22%)
M694I	Exon 10	Pathogenic	Pathogenic/Likely Pathogenic	207 (13.99%)	236 (7.91%)
M680I	Exon 10	Pathogenic	Pathogenic/Likely Pathogenic	95 (6.42%)	113 (3.79%)
A744S	Exon 10	VUS	Conflicting classifications of pathogenicity	82 (5.54%)	83 (2.78%)
R761H	Exon 10	Likely Pathogenic	Pathogenic/Likely Pathogenic	73 (4.93%)	79 (2.65%)
P369S	Exon 3	VUS	Conflicting classifications of pathogenicity	45 (3.04%)	46 (1.54%)
E167D	Exon 2	Likely Pathogenic	Pathogenic	21 (1.42%)	21 (0.70%)
K695R	Exon 10	VUS	Conflicting classifications of pathogenicity	18 (1.22%)	18 (0.60%)
F479L	Exon 5	Likely Pathogenic	Pathogenic, VUS	17 (1.15%)	17 (0.57%)
R408Q	Exon 3	VUS	Conflicting classifications of pathogenicity	17 (1.15%)	17 (0.57%)
Others				105 (7.09%)	105 (3.52%)

VUS, variants of uncertain significance.

Moreover, our results showed that E148Q was the most frequent variant in heterozygous patients accounting for 31.34% of cases (N = 267), while in homozygotes, the most detected variant was M694V (N = 65 or 36.72%). Furthermore, M694V/V726A was the most common genotype in compound heterozygous patients accounting for 20.40% (N = 92) of cases. The frequency of the variants among the total patients (N = 1,480) is represented in Table 2.

**FIGURE 2:**
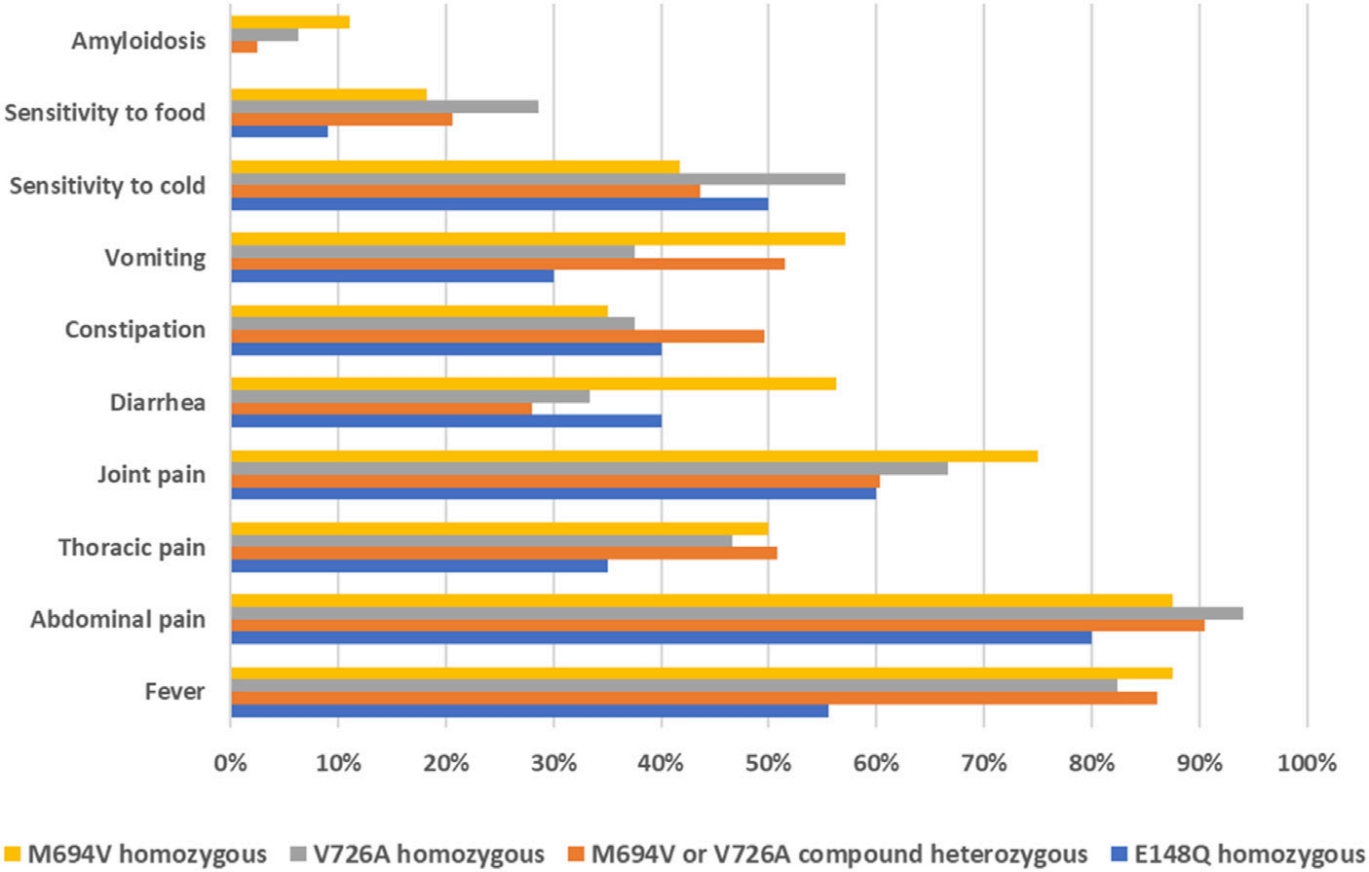
Distribution of FMF clinical manifestations based on the genotypes of affected individuals.

The most commonly reported symptoms among affected individuals were fever (78%), abdominal pain (88%), joint pain (65%), and thoracic pain (46%). Other clinical signs included diarrhea, constipation, vomiting, sensitivity to cold, and sensitivity to food. Amyloidosis was recorded in 5% of patients. Interestingly, the genotype-phenotype correlation analysis of the three most common variants revealed that patients carrying the M694V or V726A mutations, either in homozygous or compound heterozygous states, exhibited a more severe phenotype compared to those with the E148Q homozygous genotype (Figure 2). Indeed, a lower percentage of patients in the latter group reported experiencing the typical symptoms of FMF, such as fever, abdominal pain, and thoracic pain. More notably, none of the patients with the E148Q/E148Q genotype had amyloidosis.

## Discussion

This study, the largest of its kind in Lebanon and the second in the Arab World, discusses the variant and allele frequencies of the *MEFV* gene among clinically diagnosed FMF patients. Our cohort of patients showed a high frequency of pathogenic/likely pathogenic variants in exons 10, 2, 3, and 5 of the *MEFV* gene. These findings endorse the standard approach utilized at our center for the molecular diagnosis of FMF, which involves initially sequencing exon 10, followed by exons 2, 3, and 5; the remaining exons are sequenced upon request if the results from the earlier exons are negative. Benign and likely benign variants were not reported or included in this study.

The findings of this study showed that two-third of the participants were tested under the age of 20 years, which is in line with previously published data (Sohar et al., 1967; Keskindemirci et al., 2014; Yasar Bilge et al., 2018). Moreover, the proportion of infants under 1 year of age was 2.77% (N = 41) which is comparable to that observed in the previous study performed in Southern Lebanon (2.45%) (El Roz et al., 2020). In fact, diagnosis in infants at such a young age is reported to be challenging, and this is due to their lack of verbal skills that are necessary for establishing an accurate diagnosis (Keskindemirci et al., 2014). On another hand, the overall male to female ratio in our study was 1.03 and 1.12, in heterozygous and genetically confirmed patients, respectively, indicating a comparable prevalence of FMF between the two sexes. Although data about the prevalence of FMF is still elusive, most of the reports indicate that FMF affects equally males and females (Mansour et al., 2019; Jalkh et al., 2008; Pradhan et al., 2017; Duruöz et al., 2021; Gumus, 2018). Interestingly, comparison of *MEFV* frequencies with respect to the different geographical regions and ethnicities revealed that the most affected patients were from South or North Lebanon and Muslims (Shiites and Sunni). These findings align with previous reports and are most likely attributed to the high rate of consanguinity among these particular communities. Indeed, previous research conducted by Barbour and Salameh in Lebanon indicated that non-Christian individuals and those from South or North Lebanon presented high consanguinity rates, potentially because of traditional practices and social norms prevalent in these communities (Barbour and Salameh, 2009).

Additionally, family history rates of FMF were higher in the group presenting two *MEFV* mutated alleles, underscoring the importance of genetic screening in families with a known history of FMF, in order to identify at-risk individuals and enable earlier intervention for a better disease management.

Considering the significant variation in the frequency distribution of *MEFV* variants among different populations, our purpose was to compare the prevalence of the seven most common *MEFV* variants identified in this study (M694V, V726A, E148Q, M694I, M680I, A744S, and R761H) with those observed in other populations across the Mediterranean Sea, encompassing both Arab and non-Arab countries (Table 3). Notably, our study reveals several differences when compared to previous research conducted in Lebanon, especially concerning the frequency of the V726A variant. In fact, all prior studies from Lebanon (Medlej-Hashim et al., 2005; Sabbagh et al., 2008; Jalkh et al., 2008; El Roz et al., 2020) identified M694V as the sole most frequent *MEFV* variant. However, our current study shows for the first time a high prevalence of the V726A in the Lebanese population, similar to that of M694V. Such difference might be attributed to the size of the studied population and the characteristics of the sample (geographic localization, age, sex, etc.). Indeed, the Lebanese population is known to be diverse in terms of ethnicity, religious groups, and regional distribution. Unlike previous studies that involved smaller sample size (Medlej-Hashim et al., 2005; Sabbagh et al., 2008; Jalkh et al., 2008) or focused on specific regions in Lebanon (El Roz et al., 2020), our research included patients from all over Lebanon and belonging to different religious groups.

**TABLE 3:** Distribution and frequency of the seven most common *MEFV* variants among several populations, including the current study.

Country/Population	*MEFV* variants carrier rate (%)	Variants (%)						
		M694V	V726A	E148Q	M694I	M680I	A744S	R761H
Lebanon (current study)/3,167	46.7	29	29	27.8	14	6.4	5.5	5
Lebanon (Jalkh et al., 2008)/376	100	28.9	19.3	10.1	12.10	5.72	NA	NA
Lebanon (Sabbagh et al., 2008)/266	48.5	15.8	13.9	14.7	6.8	5.6	1.9	1.9
Lebanon (Medlej-Hashim et al., 2005)/558	59	30.3	19.4	8.3	12.8	7.4	1.2	3.1
Lebanon (Mansour et al., 2001)/79	67	27	20	8	9	5	0	0
South Lebanon (El Roz et al., 2020)/332	56.6	20.6	16	17.9	11.8	0	10.7	2.3
Syria (Jarjour, 2010)/153	63.4	36.5	15.2	14.5	10.2	13.2	1.8	4.4
Syria (Mattit et al., 2006)/83	89	45.8	13.9	6	9.6	4	1.2	0.6
Syria and Turkey (Gumus, 2018)/296	45.6	17	6.5	16	2.8	10	2.5	8
Palestine (Ayesh et al., 2005)/511	58.5	49	16.7	8.5	11.9	4	1.6	0.8
Jordan (Habahbeh et al., 2015)/3,359	44.4	30	20	21.5	8.3	9	3.1	0.8
Algeria (Ait-Idir and Djerdjouri, 2020)/183	47.5	14.5	0	3.3	63.2	14.5	6.2	0
Egypt (Gezery et al., 2010)/316	57.6	7.8	15.6	22.7	34	12.1	4.3	0.7
Egypt (Mansour et al., 2019)/1,387	57.2	6	15.8	38.6	18.1	9	9.3	0.2
Morocco (Belmahi et al., 2012)/120	47%	47	0	6.5	32	0	6.5	0.8
Tunisia (Chaabouni et al., 2007)/139	44%	27	5	18	13	32	3	1
Turkey (Familial Mediterranean fever, 2005)/1,090	NA	51.4	8.6	NA	NA	14.4	NA	NA
Turkey (Cekin et al., 2017)/514	45	48	12.5	18	0	15	0.5	0.9
Turkey Van province (Coşkun et al., 2015)/1,058	42.8	36.5	14.1	32.8	4.4	3.9	0.9	4.1
Turkey Hatay province (Gunesacar et al., 2014)/1,000	61.8	8	1.9	8.9	1	2.40	0.80	0.6
Greece (Konstantopoulos et al., 2003)/62	85.5	48	NA	14	NA	NA	NA	0
Italy (La Regina et al., 2003)/71	41	16	2.8	14	10	14	NA	2.8
Spain (Aldea et al., 2004)/50	64	37	3.1	16	12.5	NA	NA	NA
Iran (Bonyadi et al., 2015)/1,330	100	42.5	19	20.9	2.1	14.1	0.2	0.7
Iran (Mohammadnejad and Farajnia, 2013)/130	60	40.2	13.7	17.6	2.9	12.7	1.5	NA

The frequency distribution of the various MEFV variants shows a statistically significant difference (*p* < 0.0001) among the different studied populations, as indicated by Chi square analysis; NA: data not available.

Research conducted in other Arab countries, such as Syria (Gumus, 2018; Jarjour, 2010; Mattit et al., 2006), Palestine (Ayesh et al., 2005), Jordan (Habahbeh et al., 2015), and Morocco (Belmahi et al., 2012) indicates that, similar to Lebanon, M694V is the most prevalent variant in these populations, while the frequency of other variants vary. In fact, it has been noted that Arabs exhibit a very diverse mutational pattern influenced by geographic origin (Touitou, 2001). For instance, oriental Arabs tend to have a higher prevalence of V726A compared to Arabs from North Africa; our study further supports this observation by recording elevated rates of V726A in Lebanon for the first time. Additionally, a 2001 study suggested that M694V and V726A likely originated in the middle East region, as their prevalence among Oriental Jews falls between that of Jews from North Africa and the Ashkenazi. On another hand, if we compare the results of Arab populations (Mansour et al., 2019; Belmahi et al., 2012; Gezery et al., 2010; Ait-Idir and Djerdjouri, 2020; Chaabouni et al., 2007) with those from our study and Arab Asian populations, we observe clear differences in the prevalence of the two variants M694I and E148Q. Indeed, M694I and E148Q are highly prevalent in Algeria (49.7%) (Ait-Idir and Djerdjouri, 2020) and Egypt (38.6%) (Mansour et al., 2019), respectively. Furthermore, M680I appears to be a particularly common frequent variant within the Tunisian population, with a notable allele frequency of 32%, which is not seen in any other Arab or non-Arab populations.

Finally, our findings align with those from studies conducted in non-Arab countries situated around or near the Mediterranean sea, including Greece (Konstantopoulos et al., 2003), Italy (La Regina et al., 2003), Spain (Aldea et al., 2004), Iran (Bonyadi et al., 2015; Mohammadnejad and Farajnia, 2013), and Turkey (Familial Mediterranean fever, 2005; Coşkun et al., 2015; Cekin et al., 2017), where M694V is also the most frequent variant, with frequencies sometimes surpassing 35%. Specifically in Turkey, where the prevalence of FMF is known to be the highest (Familial Mediterranean fever, 2005), literature has shown that the allele frequency of M694V can exceed 50% in certain regions. Nevertheless, it is important to note that in Hatay Province, located in the Mediterranean region of Turkey, R202Q emerged as the most common variant (21.35%) in a sample of 1,000 clinically diagnosed patients, followed by E148Q (8.85%), while M694V ranked third at 7.95%. These findings underscore that the differences in variants frequencies can be attributed to the specific sub-populations studied as well as the geographic and socio-demographic characteristics of the participants; a conclusion that is consistent with the diverse studies conducted in Lebanon.

The analysis of the distribution of clinical manifestations based on the genotypes of the affected individuals revealed a more severe phenotype among those carrying the M694V or V726A variants. This was evidenced by the higher frequency of typical FMF symptoms, namely, fever, abdominal pain, and thoracic pain, in these patients compared to individuals with the homozygous E148Q genotype. These findings corroborate earlier studies indicating that M694V has the highest penetrance and is associated with the most severe phenotype of the disease. On the contrary, the clinical significance of E148Q is contentious, with ongoing debates regarding whether it represents a pathogenic variant with low penetrance, or merely a benign polymorphism (El Roz et al., 2020). Our genotype-phenotype correlation findings underscore the vital role of molecular diagnosis in FMF. Beyond confirming the clinical diagnosis, molecular insights are crucial for understanding symptom onset and disease severity, thereby enabling more effective management and personalized care for patients.

In conclusion, this study represents the largest analysis of *MEFV* gene variants in a Lebanese population, providing valuable insights into the molecular aspects of FMF. For the first time, we report a high prevalence of the V726A variant in the Lebanese population, which differs from previous reports from Lebanon and other regional studies. V726A, along with M694V, was associated with the most severe phenotype of the disease, in contrast to E148Q. Our results enhance our understanding of the epidemiology, genetics, and clinical presentation of FMF in Lebanon and contribute to the broader global knowledge of this disease. Additionally, they emphasize the significant role of molecular diagnosis and genotype-phenotype correlation studies in improving the clinical management of FMF patients, and in providing genetic counseling for affected patients. Further studies exploring less common variants are needed to better understand the molecular spectrum of FMF in Lebanon.

## Data Availability

The datasets presented in this article are not readily available because of ethical constraints. Study participants did not provide consent for publicly sharing all the data. Requests to access the datasets should be directed to the corresponding author, Dr Alain Chebly, at alain.chebly@usj.edu.lb.
